# Weak association of Usutu virus and haemosporidian infection in birds collected in Germany

**DOI:** 10.1016/j.onehlt.2024.100868

**Published:** 2024-08-02

**Authors:** Carolin Hattendorf, Dániel Cadar, Stefan Bosch, Norbert Becker, Lars Lachmann, Jonas Schmidt-Chanasit, Anna Heitmann, Renke Lühken

**Affiliations:** a Bernhard Nocht Institute for Tropical Medicine, Bernhard-Nocht-Straße 74, 20359 Hamburg, Germany; b Nature and Biodiversity Conservation Union (NABU), Charlottenplatz 17, 70173 Stuttgart, Germany; c Institute for Dipterology, Georg-Peter-Süß-Straße 3, 67346 Speyer, Germany; d University of Heidelberg, Grabengasse 1, 69117 Heidelberg, Germany; e Nature and Biodiversity Conservation Union (NABU), Charitéstraße 3, 10117 Berlin, Germany; f Universität Hamburg, Faculty of Mathematics, Informatics and Natural Sciences, Mittelweg 177, 20148 Hamburg, Germany

**Keywords:** Usutu virus, Haemosporidia, Plasmodium, Birds, Germany

## Abstract

The Usutu Virus (USUV) is a mosquito-borne flavivirus originated in Africa. The virus circulates in Germany since 2010. It is primarily transmitted and maintained in the natural cycle by *Culex* mosquitoes and primarily affects birds, particularly Eurasian blackbird (*Turdus merula*), leading to significant mortality. Several studies have reported a high co-infection rate of European birds with both USUV and haemosporidians. Haemosporidians are blood parasites which maintain an enzootic life cycle with birds via different arthropod vectors. This study conducted screenings of birds from Germany received through a citizen's science project for both, USUV and haemosporidians between 2016 and 2021. The prevalence of USUV reached its peak in 2018, when it was first detected throughout most parts of Germany rather than being limited to localised hotspots. Subsequently, USUV prevalence consistently declined. On the other hand, the prevalence of haemosporidians initially declined between 2016 and 2019, but experienced a subsequent increase in the following years, exhibiting a more or less inverse pattern compared to the prevalence of USUV. In 2020, a statistically significant positive association between both pathogens was found, which was also detected across all years combined, indicating if at all a weak relationship between these pathogens.

## 1 Introduction

Usutu virus (USUV) is a zoonotic flavivirus transmitted by mosquitoes. Birds serve as amplifying hosts [1]. In Europe, the primary vectors are likely *Culex pipiens* s.s. and *Culex torrentium* [2]. The virus originally emerged in Africa over 500 years ago and has been introduced into Europe several times, leading to local establishment and subsequent spread [3,4]. Currently, USUV is considered established in at least 17 European countries [5,6], including Germany [7], where it has caused several outbreaks with massive bird die-offs in recent years [8,9]. Among the affected bird species, the Eurasian blackbird (*Turdus merula*) seems to be particularly vulnerable to the virus. In 2016, areas with USUV circulation in Germany experienced an estimated 15% decline in local Eurasian blackbird populations [10]. While humans can be infected with USUV, they are considered dead-end hosts, and asymptomatic infection are reported frequently [11,12]. However, in immunocompromised individuals more severe cases were observed, including meningoencephalitis or neurological disorders like diopathic facial paralysis [13,14].

Haemosporidians are widely distributed blood parasites [15]. The most well-known representative is *Plasmodium falciparum*, which causes malaria in human and results in over 600.000 fatal cases per year [16]. Birds can not only be infected by haemosporidian species of the genus *Plasmodium*, but also by members of the genera *Haemoproteus* and *Leucocytozoon* [17]. They are transmitted between birds through pathogen genus-specific dipteran families, such as Culicidae (*Plasmodium* spp.), Ceratopogonidae and/or Hippoboscidae (*Haemoproteus* spp.), and Simuliidae (*Leucocytozoon* spp.). Studies conducted in Germany and other European countries indicated a relatively high prevalence of avian malaria infections in wild bird populations often exceeding 50% (e.g. [[18], [19], [20]]). While most bird species are considered to be well adapted to haemosporidian infections [17], recent research has challenged this notion by demonstrating significant fitness loss [21] and mortality [22] among native birds regularly exposed to haemosporidians.

Different studies suggest a positive association between USUV and haemosporidian infections in birds [[23], [24], [25]]. During the large-scale USUV outbreak in Central Europe in 2016, two independent studies reported high co-infection rates of USUV and haemosporidians in birds. In the Netherlands, half of the 16 birds infected with USUV were also infected with *Plasmodium* spp. [24]. In Belgium, out of 91 birds diagnosed with USUV, 90 were co-infected with *Plasmodium* spp. or *Haemoproteus* spp. [23]. Additionally, an earlier study from Italy in 2009 reported a co-infection with haemosporidia in 18 out of 35 USUV-positive birds [25].

Co-infections of pathogens with haemosporidians have been shown to reduce fitness and the survival probability of birds. Examples include double haemosporidian infections [26], co-infections with *Plasmodium* spp. and Bagaza virus [27], or *Plasmodium* spp. and chicken anaemia virus [28]. USUV already poses a threat to local bird populations. Therefore, it is crucial to determine whether co-circulating pathogens play a significant role in the spatial transmission risk of USUV. The interaction between avian malaria parasites and the probability of USUV infection in birds, might also help to understand the potential impact of this interaction on the spillover to humans.

As part of a dead bird surveillance programme in Germany, we conducted screenings on dead birds collected from 2016 and 2021 to detect USUV and haemosporidian infections. The results were used to analyse the association between USUV and haemosporidians.

## 2 Materials & methods

Through press releases and subsequent media coverage, citizens all over Germany were asked to contribute to the dead bird surveillance programme by sending dead birds to the Bernhard Nocht Institute for Tropical Medicine in Hamburg, Germany. They were also requested to provide information regarding the date and location of the bird's discovery, i.e. street, house number and city. Bird carcasses were shipped as post parcel (priority within 24 h or non-priority) and the delay between dead and finding of the bird are generally unclear. This surveillance programme has been in operation since the initial observation of the USUV outbreak in 2011 [7,10,29,30]. Whenever possible, samples of the heart, liver, and brain were collected from each dead bird specimen. The bird species are dominated by the European blackbirds and only data for this species are presented here.

A mix of heart, liver, and brain tissues of each bird specimen were homogenised and subsequently subjected to DNA/RNA extraction using KingFisher™ Flex Magnetic Particle Processor (Thermo Fisher Scientific, Waltham, MA, USA) with the MagMAX™ Pathogen ribonucleic acid/DNA Kit (Thermo Fisher Scientific, Waltham, MA, USA).

USUV screening was conducted with a modified pan-flavivirus reverse transcription PCR [29]. For haemosporidian screening, we used a nested PCR protocols developed by Bell et al. [31]. This included a nested PCR targeting the cytochrome *b* gene of *Plasmodium* spp. and *Haemoproteus* spp., and a nested PCR for the same gene of *Leucocytozoon* spp. All PCR amplicons were sent to LGC Genomics (Berlin, Germany) for Sanger sequencing. The sequences were processed with Geneious 7.1.9 (Biomatters, Auckland, New Zealand) and compared to available sequences in the GenBank [32] using the basic alignment search tool (BLAST) in the GenBank DNA sequence database [33]. Haemosporidian sequences were additionally compared to sequences in the MalAvi database [34].

The data were analysed using R software [35] with the packages raster, sp., zoo, and ggplot2. The association of co-infection cases was tested using a Pearson's χ^2^-test with Yates' continuity correction and temporal correlation was analysed via a Pearson's product-moment correlation test.

## 3 Results

**Fig. 1:**
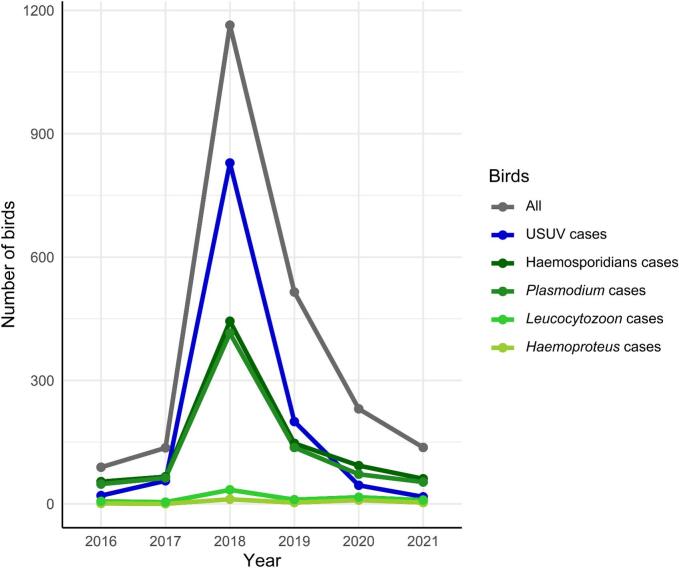
Number of birds and infections between 2016 and 2021. Haemosporidian cases are shown accumulatively and separated per genera.

This study includes birds received between 2016 and 2021 (*n* = 2272). 89 birds were screened in 2016, 136 birds in 2017, 1164 birds in 2018, 515 birds in 2019, 231 birds in 2020, and 137 birds in 2021 (Fig. 1).

**Fig. 2:**
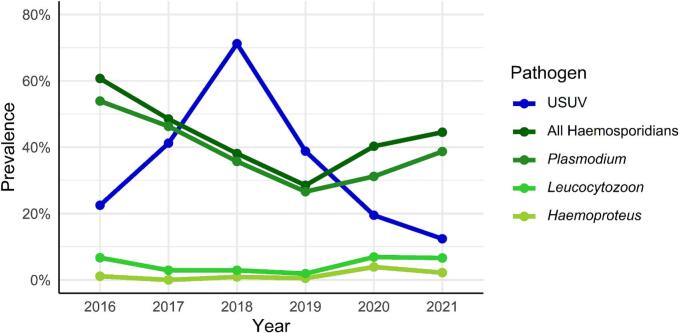
USUV and haemosporidian prevalence in investigated birds. The prevalence for haemosporidians are shown accumulated and separated for each genus.

The overall USUV-prevalence rose from 22.5% in 2016 to 41.2% in 2017 (Fig. 2). In 2018, we observed the biggest USUV outbreak so far, with a sharp increase of USUV prevalence to 71.2%. The massive outbreak was also reflected in the total number of birds submitted, which increased more than 8-fold from 2017 to 2018 (Fig. 1). Subsequently, the prevalence of USUV decreased to a level similar to that of 2017 (Fig. 2), but USUV cases were still detected across all regions of Germany. In the subsequent years, the prevalence of USUV continued to decline even further, reaching 19.5% in 2020 and 12.4% in 2021. These cases remained scattered throughout the entire country.

Haemosporidian infected birds were found throughout the country in all years (Fig. 1). The highest prevalence was detected in 2016 with 60.7% (Fig. 2). Afterwards, the prevalence decreased yearly by roughly 10% until it reached its lowest point in 2019 with 28.5%. 2018 and 2019 were the only years in which the haemosporidian prevalence was exceeded by USUV prevalence. The following years, the haemosporidian prevalence rose again to 40.3% in 2020 and 44.5% in 2021. The vast majority of haemosporidian infections were caused by *Plasmodium* spp. We identified only a few cases of *Haemoproteus* spp. and *Leucocytozoon* spp. each year.

**Fig. 3:**
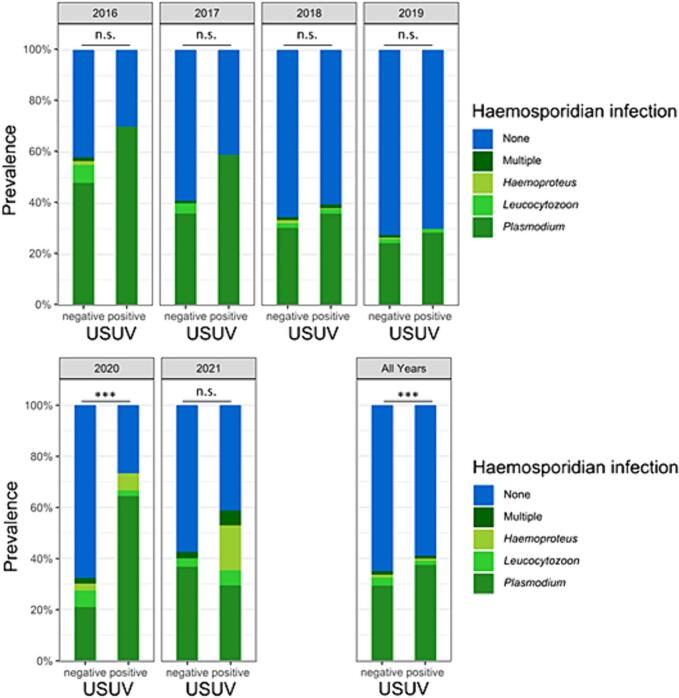
Dispersion of haemosporidian infected and uninfected birds for USUV positive and negative birds for the years 2016–2021. Single haemosporidian infection are shown by genus, infections of multiple genera in single birds are shown as “Multiple”. The total number of USUV-positive/USUV-negative were 20/69 for 2016, 56/80 for 2017, 829/335 in 2018, 200/315 in 2019, 45/186 in 2020, and 17/120 in 2021.

In all six investigated years, USUV-positive birds were more often co-infected with at least one haemosporidian species than USUV-negative birds (Fig. 3), although this association was only statistically significant for the year 2020 (*p* = 1.103*10^−6^). Nevertheless, looking at the whole period of six years, this association was found highly significant (*p* = 4.108*10^−3^).

Most haemosporidian cases were caused by *Plasmodium* spp. and there was a statistically strong association of USUV and *Plasmodium* spp. in 2020 (*p* = 2.09*10^−7^) and for all years together (*p* = 1.6*10^−4^) as well. Additionally, there was a slight association in 2017 (*p* = 0.02192). The other years showed no significant association. There were only 27 *Haemoproteus* spp. cases of all 2272 birds screened and there was no statistically significant association with USUV for any year, except for 2021 (*p* = 1.648*10^−4^). However, there were only 3 *Haemosporidian* spp. infections in 2021, so the chi-square test might not be reliable. An association of *Leucocytozoon* spp. and USUV was only found for all years (*p* = 0.02094) and not for any individual year.

## 4 Discussion

Since its initial detection in German in 2010, outbreaks of USUV had been initially limited to South-West Germany, gradually expanding their circulation area towards the North [29,36]. However, in 2018, a rapid and wide emergence of USUV was observed throughout Germany and other western European countries [5,8], resulting in the largest USUV outbreak reported so far. The number of dead birds submitted to our dead bird surveillance programme increased more than 8-fold, suggesting a significant mortality rate among the German bird population due to USUV infections. In the following years, USUV prevalence in the submitted dead birds continuously decreased to only 12.4% in 2021. This suggests that the major outbreak was driven by the expansion of USUV into new regions, where previously unexposed bird populations were highly susceptible to the virus.

In contrast to USUV, haemosporidians are not a re-emerging pathogen among birds, but have been circulating in Germany and Europe for a very long time [17]. Numerous studies consistently demonstrate the presence of haemosporidian infections in birds throughout Europe (e.g. [37,38]). Therefore, it was not surprising to find circulation of haemosporidia across Germany in all years. However, it is noteworthy that the prevalence consistently decreased from 2016 to 2019, only to rise again in 2020 and 2021. This trend appears to be an inversion to the USUV prevalence pattern, although the peak of USUV prevalence was one year prior (2018) to the lowest point of haemosporidian prevalence (2019). This might suggest that the spread of USUV and subsequent decrease in local bird populations [10] may be one of the primary factors influencing the haemosporidian prevalence. There have been some studies indicating that haemosporidian prevalence may be linked to host abundance and density, although this appears to vary for individual haemosporidian species [39,40]. As USUV circulation decreased and the bird populations recovered, the haemosporidian circulation increased once again.

Varying prevalence of the haemosporidian genera are reported regularly. For instance, Lüdtke et al. discovered in 2011 that 99.4% of diagnosed blood parasite infections in German passerines were caused by *Haemoproteus* spp., with an overall prevalence of 39% [41]. Another study by Schumm et al. conducted on German passerines in 2015, 2017, and 2018 identified *Leucocytozoon* spp. as the most prevalent genus, with 71% of tested birds infected, compared to a prevalence of 13% for *Plasmodium* spp. and 31% for *Haemoproteus* spp. [42].

In contrast, our study revealed *Plasmodium* spp. to be the predominant causative agent in the vast majority of haemosporidian infections (91.1%). It is important to consider that the discrepancies in findings among studies can arise from different study design. It is in the nature of such a citizen science project that the birds are predominantly sent from urban areas, which generally show a high density of mosquitoes as vectors of *Plasmodium* spp., but low densities of Ceratopogoniae/ Hippoboscidae or Simuliidae as vectors of *Haemoproteus* spp. and *Leucocytozoon* spp., respectively (REF). In addition, although all studies focused on German passerines, the bird species in focus varied. This study analysed predominantly Eurasian blackbirds, which were tested neither in the studies by Lüdtke et al. [20] nor by Schumm et al. [42]. Furthermore, like most studies, they tested blood samples rather than organ samples, as done in this study. Haemosporidians are considered to be primarily blood-parasites and testing the blood has been shown to be a sensitive method [43]. Nonetheless, it has been shown that haemosporidians can be diagnosed using organ samples as well, because after an initial replication period in the blood stream, haemosporidians often enter latent, exoerythrocytic stages [44]. However, this study used bird carcasses that have been potentially left at room temperature for several days during shipping and the potential effects of this handling procedure on the detection sensitivity are not fully understood. Furthermore, it is worth noting that the nested PCR for the genus *Leucocytozoon* also amplified members of *Plasmodium*. It has been previously observed that these PCR assays tend to amplify the most abundant haemosporidian DNA [45]. This limited our study to detect haemosporidian-double infections and their impact on an additional USUV infection.

USUV-infected birds were more likely to have co-infections with haemosporidians compared to USUV-uninfected birds, although this difference was not statistically significant for most individual years. These findings align with other studies, which reported different results. Studies from Italy (2011) and Netherlands/Belgium (2016) reported a significant positive association between both pathogens [[23], [24], [25]]. More recent studies from the Netherlands confirmed regular co-infection of European blackbirds with USUV and *Plasmodium* spp. (2016–2018, 2016–2020) [46,47]. It was also demonstated that the same organs are affected by both pathogens, but the severity of lesions in multiple organs (liver, spleen, heart, brain, and lungs) is increased in co-infections. In contrast, a study from Austria in 2018 found no association [48]. Environmental factors such as climate or vector density might have a stronger influence on circulation patterns of both pathogens, thereby masking a potential association in certain years and areas. Birds, particularly the Eurasian blackbird, can experience severe illness as a result of USUV infection [46], which can leave them weakened and less mobile. This reduced mobility might make them more susceptible to haemosporidian-transmitting vectors [49] and their already compromised health may increase the risk of haemosporidian infection. While native birds are generally considered to be well adapted to haemosporidians and often do not develop severe illness [17], recent studies suggest that haemosporidian infection can have significant negative fitness consequences [21,22]. The long-term effects of chronic manifestations, which occur after the peak of parasitaemia have often been overlooked in the past [50]. Therefore, birds with haemosporidian infections may be more susceptible for an additional USUV infection and vice versa. This fits in with the fact that it has recently been shown that co-infections with USUV and *Plasmodium* spp. leads to higher lesion severity in European blackbirds compared to single-infections [47].

## 5 Conclusion

There appears to be a weak correlation between USUV and haemosporidian infections in birds from Germany, although the exact nature of this relationship remains unclear. We observed that as the prevalence of USUV increased sharply, the prevalence of haemosporidians decreased and vice versa. However, additional studies including the environmental parameters, e.g. land-use or mosquito abundance, driving the infection risk are required to gain a deeper understanding of the causal relationship between USUV and haemosporidians.

## Funding

This work was financially supported by the 10.13039/501100005908German Federal Ministry of Food and Agriculture (BMEL) through the 10.13039/501100010473Federal Office for Agriculture and Food (BLE), with the grant number FKZ 2819113519 and the 10.13039/501100002347Federal Ministry of Education and Research of Germany (BMBF) under the project NEED (grant number 01Kl2022).

## Ethics declarations

The ethcis committee of the lead organization confirmed that ethics approval was not required.

## CRediT authorship contribution statement

**Carolin Hattendorf:** Data curation, Formal analysis, Writing – original draft. **Dániel Cadar:** Formal analysis. **Stefan Bosch:** Data curation. **Norbert Becker:** Data curation. **Lars Lachmann:** Data curation. **Jonas Schmidt-Chanasit:** Conceptualization, Writing – review & editing. **Anna Heitmann:** Conceptualization, Formal analysis. **Renke Lühken:** Conceptualization, Data curation, Formal analysis, Writing – review & editing.

## Declaration of competing interest

The authors declare no conflict of interest.

## Data Availability

Data will be made available on request.
